# Congenital Dislocation of Both Hip Joints*Read before the Buffalo Medical Club and patient presented.

**Published:** 1884-04

**Authors:** Herman Mynter

**Affiliations:** Surgeon to Buffalo General Hospital


### Clinical Reports.

### Congenital Dislocation of Both Hip Joints.*

By Herman Mynter, M. D.,

Surgeon to Buffalo General Hospital.

The patient, a little girl, 8 years of age, was brought to my office a couple of months ago for treatment for spinal curvature, her parents stating that in their opinion the child instead of growing became smaller as the years passed. She had never complained of pain or sustained any injury, and I am free to state that I for some days did not discover the real nature of her complaint, and was inclined to look upon it as an unusual relaxation of the ligaments of the spine and muscular weakness. So much more as I never had seen a similar case, and expected that a patient with a double dislocation of the hips would be utterly unable to walk so well or have so free abduction and rotation outwards of the hips as she has. In regard to previous history, I may state that nothing unusual happened to the mother during her pregnancy with this child. The confinement was normal, the head presenting; no instruments were used, a midwife being in attendence. The child was small during its infancy, but did not try to walk before it was 3 years old. No physician was ever consulted. It has since been able to walk and run and jump, does not easily tire, being fatigued scarcely more than any other children of the same age, and is seemingly as healthy a little girl as could be wished. The parents consulted me, as I said, about the spinal curvature. Upon examination a curious picture presents itself. The pelvis is considerably inclined forwards, the buttocks abnormally projecting upwards and backwards, and we see a lordosis of the lumbar vertebræ, with compensating curve of dorsal vertebræ. The pelvis seems abnormally broad, and the trochanter major more prominent than usual and approaching the crest of the ileum,

* Read before the Buffalo Medical Club and patient presented.

The most notable feature is the great length of the upper as compared with the inferior part of the body. The muscles of the lower extremities are not so much affected in this case as usual, the thighs have an inward direction, and by walking would strike each other, if not on account of the peculiar gait. The feet are bent slightly outward and slight flat-foot is present. The gait is the peculiar rolling motion with "double lameness," characteristic of this lesion. It reminds me of the walk of a duck. When the child is lying down, the lordosis disappears by traction upon the legs, and the same occurs when you let the child sit down. She then sits on the ischial tuberosities, and having then no cause for tilting the pelvis, the lordosis disappears immediately.

By examining the motion of the hips, we find them perfectly free and easy, almost too free, with the exception of abduction in the flexed condition and rotation outwards. These two movements are not as free as usual, and if carried too far, are prevented by contraction of the abductor muscles. By measuring we find that the right trochanter is two inches above Nelaton's line, which measurement by traction may be reduced to one inch. On the left side the trochanter is about one inch above Nelaton's line; by traction this measurement is reduced to one-half inch. By traction the legs may be elongated one inch. The distance from the crest of the ileum to the trochanter may be increased one inch by traction. The heads of the femur are felt by rotation on the ileum, easiest when the child is lying on her abdomen. Congenital dislocations of the hip are, if not very rare, certainly uncommon. They are found oftener in girls than in boys. Of forty-five cases collected by Dupuytren, thirty-nine were females. The majority are double dislocations, only seven out of forty-five being single. The affection is by some believed to be hereditary. This belief rests on a statement of Dupuytren, which, according to Holmes, is insufficiently supported. The direction of the dislocation is upwards and outwards on the ileum; dislocations upwards and forwards have never been found except in fœtal monstrosities. The most interesting point, and that about which the authors differ most, is the etiology and pathology. The most important point to decide is, whether these cases are really dislocations, or, as Sayre and Bryant especially maintain, malformation of the acetabulum, namely, a non-fusion of the three bones which enter into its construction, or, as Breschet calls it, arrest of development. Bryant mentions a couple of interesting anatomical preparations, which have a distinct bearing upon this point, one in which the cause was want of depth from incomplete formation of the acetabulum (Cruveilhier's Pathol. Anatomy), another from the Middlesex Hospital Museum, in which there is practically no acetabulum but a strong capsule and a well-developed head of the femur without a ligamentum teres. Sayre objects to the name dislocation, as there never has been any luxation. Other authors believe the cause to be disease within the articulation of the fœtus, especially synovitis. Holmes acknowledges that effusion taking place within the joint, dislocation might occur, but thinks that no proof has really been given that this has absolutely taken place. This view has been maintained by Sedillot and Malgaigne and was originally proposed by Ambrose Paré. It seems to me that if this view was correct we might, at any rate occasionally, find some traces of inflammation, some tenderness of the parts, the more that a synovitis, with an effusion great enough to produce a dislocation, surely is a very serious lesion and could not be expected to disappear perfectly within one-half year. It is a fact, that there is never any pain present at birth. Carnochan considers the defect a result of active morbid muscular retraction, on account of a perverted or disturbed condition of the excito-motor apparatus of the medulla spinalis. It sounds very fine, but I am unable to understand, what should produce this disturbed condition, or, if really present, why it should produce dislocation in the fœtus, but never in a living child or adult. External violence has been mentioned as the cause since the time of Hippocrates. That it may produce fracture is well proved, and that the accoucheur might, by manipulation and traction on the limbs in breech presentation, produce it, is within the realm of possibilities, but, as Bryant puts it, no evidence has been published to support this view. In no case has he been able to learn that a breech presentation took place. He heard, in the majority, that a natural birth occurred. My private opinion is, that the want of pain in congenital dislocation speaks absolutely against this view, and I believe that even an infant, immediately after birth, when it is handled so much, would make it so lively in a house, if it had a single, not to mention a double dislocation, that the cause would be discovered. But it is a fact, that the lesion is not discovered before the child commences to walk. At birth, as Holmes says, it passes unobserved, and there is nothing to attract attention to the displacement; it is painless, or, if not entirely painless, it is not observable, for the direction of the limb is not sensibly altered and the motion of the head of the bone is free.

In regard to pathology, it is asserted by Hamilton that the head of the femur may be changed in form and consistence, or be altogether wanting. The pelvic bones are usually more or less deformed; the acetabulum may be entirely deficient, or may present itself as an irregular, bony protuberance, without cartilage, fibro-cartilage or ligaments. Sometimes it exists as an oval or triangular cavity, which is expanded at its superior and posterior margin into a distinct fossa where the head of the femur occasionally rests. A new cavity is formed, usually upon the side of the pelvis, which is shallow and without an elevated margin. The new socket is often lined with a true periosteum and synovial membrane. Sometimes the surface is hard and polished, like ivory. The head of the femur, having escaped from its original capsule, rests in the new socket constantly, or the head remains within its capsule, and may be observed to play backwards and forwards between the two sockets; or, the head and neck being absorbed and the capsule remaining entire, the latter is converted into a long, narrow sack, or into a ligamentous cord. In this case no new socket is formed.

In regard to diagnosis, it is mentioned that this affection may be mistaken for coxitis. It is scarcely necessary to point out the difference in the symptoms of these two affections. It is more probable that it may be confounded with infantile paralysis.

Can anything be done to remedy this affection? Different authors mention different kinds of treatment, according to whether the dislocation is single or double, and whether it be discovered soon after birth, or years afterwards. The whole question, it seems to me, depends upon whether it really is a dislocation, or, what seems more probable, a malformation. If there is no acetabulum, or only an imperfectly developed acetabulum, in which there has been no luxation, as Sayre puts it, it seems useless to attempt a reduction. In a single dislocation it seems to me an attempt ought to be made as soon as discovered. There is in these cases at least a possibility that the dislocation may have been produced accidentally. Another point, having a distinct bearing upon the treatment, is whether we can decide if the head of the femur is inside the capsule or not. If inside the capsule, the looseness must necessarily be greater, as the head can play backwards and forwards as far as the capsule will allow it. If outside the capsule, an inflammation, with formation of osteo-phytes and a new joint will necessarily be the result, and consequently a considerable decrease of the mobility. Ought we not, therefore, to make some attempts to open the capsule and bring the head of the femur in direct contact with the ileum? This process is in reality what Guerin proposes: "The means which I adopt," he says, "are based upon a recognition of the processes which nature employs for the attainment of the same purpose. I have shown that the essential condition of the formation of artificial cavities is perforation of the articular surface and the placing in contact of the luxated extremity with an osseous surface, and that the condition of maintenance is the intimate adherence of the border of the rent with the circumference of the new cavity." To do this he practices scarifications of the capsule down to the bone to which it is attached. By this means the dislocated extremity is placed in immediate contact with the bony surface, adhesion and fusion of the scarified points with the corresponding points takes place, and at last deep scarifications are made for the same purpose around the head, that it, by the fibro-cellular adhesion, may be imprisoned. Finally, when the fibro-cellular adhesions are supposed to be sufficiently solid, movements are begun, to develop a cavity for the head. I consider this whole process perfectly rational and feasible. If anything should be done, this is the only remedy. Bandages surrounding the pelvis can scarcely do any good, and Sayre's extension apparatus, it seems to me, is worse than useless. Necessarily it will take a long time to accomplish such a result. My little patient here walks, as you will see, so well that I scarcely could recommend a treatment which may take years to carry out. I do not in her case recommend anything except, when the time comes, to adopt a sedentary occupation, although from the great looseness of the joints I am inclined to believe that the capsula are intact, and that therefore this case would be a suitable one for Guerin's operation.

